# Cardiovascular health effects following exposure of human volunteers during fire extinction exercises

**DOI:** 10.1186/s12940-017-0303-8

**Published:** 2017-09-06

**Authors:** Maria Helena Guerra Andersen, Anne Thoustrup Saber, Peter Bøgh Pedersen, Steffen Loft, Åse Marie Hansen, Ismo Kalevi Koponen, Julie Elbæk Pedersen, Niels Ebbehøj, Eva-Carina Nørskov, Per Axel Clausen, Anne Helene Garde, Ulla Vogel, Peter Møller

**Affiliations:** 1 Department of Public Health, Section of Environmental Health, University of Copenhagen, Øster Farimagsgade 5A, DK-1014 Copenhagen K, Denmark; 2 The National Research Centre for the Working Environment, Lersø Parkalle 105, DK-2100 Copenhagen Ø, Denmark; 3 Danish Technological Institute, Teknologiparken, Kongsvang Allé 29, DK-8000 Aarhus C, Denmark; 4 Department of Public Health, Section of Social Medicine, University of Copenhagen, Øster Farimagsgade 5A, DK-1014 Copenhagen K, Denmark; 5 Department of Occupational and Environmental Medicine, Bispebjerg Hospital, Bispebjerg Bakke 23, DK-2400 Copenhagen, NV Denmark; 6 Department of Micro- and Nanotechnology, Technical University of Denmark, DK-2800 Kgs. Lyngby, Denmark

**Keywords:** Cardiovascular disease, Firefighter, Ultrafine particles

## Abstract

**Background:**

Firefighters have increased risk of cardiovascular disease and of sudden death from coronary heart disease on duty while suppressing fires. This study investigated the effect of firefighting activities, using appropriate personal protective equipment (PPE), on biomarkers of cardiovascular effects in young conscripts training to become firefighters.

**Methods:**

Healthy conscripts (*n* = 43) who participated in a rescue educational course for firefighting were enrolled in the study. The exposure period consisted of a three-day training course where the conscripts participated in various firefighting exercises in a constructed firehouse and flashover container. The subjects were instructed to extinguish fires of either wood or wood with electrical cords and mattresses. The exposure to particulate matter (PM) was assessed at various locations and personal exposure was assessed by portable PM samplers and urinary excretion of 1-hydroxypyrene. Cardiovascular measurements included microvascular function and heart rate variability (HRV).

**Results:**

The subjects were primarily exposed to PM in bystander positions, whereas self-contained breathing apparatus effectively abolished pulmonary exposure. Firefighting training was associated with elevated urinary excretion of 1-hydroxypyrene (105%, 95% CI: 52; 157%), increased body temperature, decreased microvascular function (−18%, 95% CI: -26; −9%) and altered HRV. There was no difference in cardiovascular measurements for the two types of fires.

**Conclusion:**

Observations from this fire extinction training show that PM exposure mainly occurs in situations where firefighters removed the self-contained breathing apparatus. Altered cardiovascular disease endpoints after the firefighting exercise period were most likely due to complex effects from PM exposure, physical exhaustion and increased core body temperature.

**Electronic supplementary material:**

The online version of this article (10.1186/s12940-017-0303-8) contains supplementary material, which is available to authorized users.

## Background

Firefighters have high risk of on-duty death due to cardiovascular diseases, whereas the life time risk is similar to the general population [1]. It has been shown that deaths from coronary heart disease were most frequent among firefighters who were actively engaged in suppressing fires, whereas those with non-emergency duties had the lowest mortality among on-duty firefighters [2]. The excess mortality has been attributed to various factors such as smoke, physical exhaustion, hyperthermia, dehydration and mental stress. Controlled studies of 3 h during fire extinction showed that firefighters had decreased left ventricular contractility and stroke volume, tachycardia and increased microvascular vasodilation within the first 30 min after cessation of the activities [3, 4]. Several studies have demonstrated that exposure to heat, associated with increased body temperature, increases the peripheral arterial compliance, shear stress and blood flow [5, 6]. Exercise also increases the body temperature and evokes a number of hemodynamic changes, including vasodilation [7]. Above all, these results demonstrate an immediate and possibly transient effect of exercise and increased body temperature on the cardiovascular physiology.

Exposure to particulate matter (PM) from combustion of carbon-based materials such as fossil fuels is associated with increased risk of morbidity and mortality of cardiovascular diseases [8]. Firefighters may be exposed to PM when they remove their self-contained breathing apparatus while not actively engaged in fire suppression activities. Bystander exposure to smoke can therefore occur and diesel exhaust from fire trucks or pumps operated by firefighters may constitute additional sources of PM exposure. A meta-analysis of epidemiological studies has shown an inverse relationship between exposure to particulate air pollution and heart rate variability (HRV) [9]. Likewise a number of studies have documented associations between exposure to PM and cardiovascular disease endpoints such as vasomotor dysfunction and progression of atherosclerosis in animal models and humans [10, 11].

The chemical composition of the smoke varies substantially from one fire to another. Fires in urban settings typically give rise to very complex mixtures because of the combustion of household equipment, whereas combustion of wood can be considered as a more “clean” type of smoke. Studies on controlled exposure to wood smoke have indicated little effect on microvascular vasomotor function [12, 13], whereas HRV was decreased [14]. To the best of our knowledge, no studies have assessed biomarkers for cardiovascular disease after controlled exposure to more complex fuels than wood, such as plastic or household materials.

The aim of the present study was to assess whether firefighting activities, using appropriate personal protective equipment (PPE), were associated with cardiovascular effects in young subjects training to become firefighters. The subjects participated in smoke diving exercises to supress wood fires with or without additional items that occur in “real” fires (i.e. electrical cords and mattresses). Markers of cardiovascular function and risk factors included vasomotor function measurements by reactive hyperemia index (RHI) and cardiac autonomic nervous system regulation by HRV. Personal exposure to polycyclic aromatic hydrocarbons (PAH) was assessed by urinary excretion of 1-hydroxypyrene (1-OHP), which is a widely used biomarker of exposure to combustion products in environmental and occupational settings [15]. Biomarkers of cardiovascular risk obtained after the firefighting exercise were compared to control measurements performed 2 weeks before and 2 weeks after the firefighting course, respectively.

## Methods

### Subjects

The subjects were healthy conscripts who participated in a rescue specialist educational course, a nine-month education under the Danish Emergency Management Agency in 2015 and 2016. Self-reported pregnancy, smoking, and drug or alcohol misuse were exclusion criteria. Fifty-four subjects were enrolled in the study in four different campaigns. One female subject dropped out of the education and cardiovascular endpoints were not measured from additional 10 subjects for logistic reasons (5 subjects in each of the campaigns 3 and 4). Consequently, the final study population consisted of 32 males and 11 females. The subjects were recruited from four consecutive training classes (campaigns): campaign 1) covered 8 conscripts in the summer; 2) 11 conscripts, autumn; 3) 17 conscripts, winter; and 4) 17 conscripts, spring. Table 1 shows the characteristics of the subjects. The distribution of female subjects between campaigns varied from 17 to 36%. The age of the participants varied from 18 to 26 years. Seventy-two percent of the subjects had a body mass index (BMI) between 18.5 and 24.9 kg/m^2^ and 28% of the subjects had BMIs between 25 and 30 kg/m^2^.

**Table 1:** Characteristics of the subjects

Characteristic	Male (*n* = 32)	Female (*n* = 11)	Total (*n* = 43)
Age (years)	21.0 ± 1.3	21.5 ± 2.1	21.1 ± 1.6
Height (cm)^a^	181.4 ± 6.7	172.1 ± 3.2	179.0 ± 7.2
Weight (kg)^a^	78.3 ± 11.4	67.6 ± 10.0	75.6 ± 11.9
BMI (kg/m^2^)	23.7 ± 2.6	22.8 ± 3.0	23.5 ± 2.7
Subjects with allergies (n)^a^	10	3	13
cBL.HR (bpm)	65.8 ± 6.6	66.9 ± 7.4	66.0 ± 6.7

*BMI* body mass index, *cBL.HR* average baseline heart rate from the two control measurements. Values are number or mean ± SD

^a^Self-reported information

### Study protocol

The design was a human exposure study, where the participants were studied in three exposure scenarios, serving as their own controls. In each campaign, the blood sampling and physiological measurements after each exposure scenario were conducted at the same time of the day, separated by around 14 days with the exception of campaign 3 where only 7 days separated the second and third exposure scenario due to the Christmas holiday. During the first exposure scenario, subjects were in a classroom receiving theoretical information. During the second exposure scenario, the subjects participated in a 3-day smoke diving training program with various types of activities in a constructed firehouse and in a flashover container. The exercises increased in complexity as the participants acquired skills and they were wearing full PPE, including a self-contained breathing apparatus. In the third exposure scenario, the subjects were having another module component of their education unrelated to firefighting. The first and third scenarios were control measurements, whereas the second period was the exposure situation. We designed two different types of fires. The subjects supressed fires of standard wooden EUR pallets in absence (campaign 1 and 2) or presence (campaign 3 and 4) of foam mattresses and electrical cords. New material (one-third of a mattress and 2 m electrical cord) was added to the fires as each team of smoke divers entered the building. In total, during each day of the 3-day smoke-diving course, 6 mattresses and 20 m of electrical cords were burned. The foam mattresses were purchased in IKEA; they consisted of polyurethane (28 kg/m^3^) with a cover fabric (64% polyester and 36% cotton) and the weight of each mattress was 6 kg. A recycling station delivered the electrical cords.

### Exposure assessment

The smoke exposure was assessed with various stationary and person-borne equipment for PM measurements that measured either the particle number or mass concentrations. The supplement contains further description of the exposure setting, including type and location of PM monitors. Personal exposure to PM was assessed immediately before, during and immediately after the fire extinction exercise for 3 subjects in the first campaign. It was not possible to obtain personal PM exposure for all subjects due to a limited number of personal monitors. We therefore focussed on determining whether PM exposure occurred when the subjects were wearing PPE, including self-contained breathing apparatus. We used the urinary excretion of 1-OHP as a biomarker of PAH exposure, whereas PAH is used as an exposure marker of PM and smoke. The subjects delivered morning urine samples on the measurement day for the control measurements and on the day after the exposure situation. The half-life of 1-OHP is 6–35 h [16], thus the 1-OHP measurement captures the exposure period, although exposures closest ﻿to the sampling contributes the most. Reverse-phase HPLC was used for the quantitative measurement of 1-OHP in urine using a previously published method [17]. We standardized for diuresis with the concentration of creatinine as used in other studies [15].

We assessed the impact of fire-related activities on the body temperature in an auxiliary experiment conducted during a smoke diving module course in 2016. The subjects performed smoke-diving exercises or acquired skills in a flashover container. Body temperature was recorded before, immediately after, and more than 20 min after fire-related activities using an ear thermometer (ThermoScan® 7, Braun GmbH, Kronberg, Germany). Two different activities were monitored: fire-suppression in the firehouse (7 to 10 min inside the firehouse with suppression or rescuing tasks to perform) and flashover container (30 min sitting inside a container with fire). It was not possible to organize a stringent exposure scenario due to logistic implications of the exercise, as some participants had to do fire extinction exercises several times or they hurried on to other exercises.

### Cardiovascular measurements

RHI and HRV measurements were primary outcomes, which were measured non-invasively using the portable EndoPAT2000 (Itamar Medical Ltd., Israel) as previously described [18]. Briefly, finger-mountable pneumatic sensors were placed on the index fingers measuring pulse volume changes through three test stages: a baseline recording (6–7 min), a brachial arterial occlusion of one of the arms, induced by inflation of a blood pressure cuff to a supra-systolic pressure (5 min), and a post-occlusion recording of the induced reactive hyperemia response (5 min). Blood pressure measurements were done with a single measurement using one aneroid sphygmomanometer, before the peripheral arterial tonometry (PAT) measurement. From the baseline recording, the EndoPAT device determines the HRV based on measurement over 5 min. The HRV results include time domain measures (SDNN, pNN50 and RMSSD), high (HF) and low frequency (LF) components as well as the LF/HF ratio. Additionally the device determines the baseline heart rate (BL.HR) and the augmentation index (AI). All the measures were done in a quiet room with the subjects resting in a seated position. The measurements in the second exposure scenario were carried out between 20 min to 3 h after cessation of the fire extinction exercise.

### Statistical analysis

We used R statistical language and the package *lme4* [19] to perform a linear mixed effects analysis of the relationship between the cardiovascular endpoints and exposure. As fixed effects, we used factorial variables of exposure (before/exposure/after) and sex (male/female) and continuous variable of BMI (without interaction terms) into the model. The exposure term in the statistical analysis was either exposure period (i.e. one exposure and two non-exposure periods within each campaign) or type of fire (i.e. wood or wood with mattresses and electrical cords). Inclusion of campaign or the type of fire in the statistical analysis using the exposure period as predictor did not alter the size of the exposure-outcome relationship; thus we have reported results that have not been adjusted for effects related to campaigns. As random effects, we used by-subject intercepts. *P*-values were obtained with the function *glht* from *multcomp* [20]. The percent changes were obtained by dividing the estimate change with the intercept value from the mixed model graph line and multiplying with 100. As the RHI was expressed on a logarithmic scale, the percent change was obtained directly from the effect estimate using the expression: (exp^estimate^ − 1)*100. The biomarker of exposure was also analysed with the same mixed model function, using the creatinine-adjusted urinary 1-OHP concentration, sex and BMI as fixed effects. The analysis of the association between the fire extinction exercise and urinary excretion of 1-OHP demonstrated a skewed distribution of residuals. A cubic root transformation of the data and removal of one outlier did not change the statistical significance of the association; thus, we have reported the statistics of the non-transformed data. Welch t-test was used to compare the difference in means of effect change between exposed and unexposed scenarios between the different types of fire. Paired t-test was used to compare the mean body temperature difference between different exposure conditions. *P*-values <0.05 were considered statistically significant. Since many of the assessed biomarkers are inter-dependent, correction for multiple testing was not performed.

## Results

### Exposure to particulate matter

The PM exposure assessment showed that the PPE with the self-contained breathing apparatus very efficiently protected the conscripts from PM exposure by inhalation during fire-suppression activities. The mean particle number concentrations in the inhalation zone inside the self-contained breathing apparatus during fire suppression activities were less than 1000 particles/cm^3^ (Additional file 1: Table S2). We were unable to assess PM levels in the fire room, but at the floor landing above the fire extinction exercises, the total PM mass concentration was 32 mg/m^3^. The subjects were exposed to higher particle number concentrations in situations when they were not wearing the self-contained breathing apparatus. This occurred when they received instructions or feedback at locations that were considered as “safe zones”. The mean aerosol particle number concentrations in the inhalation zone varied substantially among the subjects when they were not wearing the self-contained breathing apparatus (50,000–250,000 particles/cm^3^). Further information on the exposure assessment is available in the supplemental material.

### Urinary excretion of 1-hydroxypyrene

**Fig. 1:**
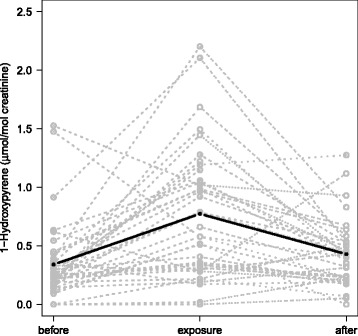
Creatinine-adjusted urinary concentration of 1-hydroxypyrene in three exposure scenarios (before and after as control measurements, and exposure measurement). Grey symbols and dashed lines are individual results in each subject. Black line is a graphical output of the mixed effect model

﻿Figure 1 shows the creatinine-adjusted urinary 1-OHP concentrations in the three exposure scenarios (control measurement before, exposure and control measurement after). Results from 6 males were excluded due to missing data for the exposure measurement (*n* = 5) or for both control measurements (*n* = 1). The exposure during the fire extinction exercise increased the urinary excretion of 1-OHP by 105% (95% CI: 52,157%) based on the mixed effects model. The association was especially driven by campaign 2 (Additional file 1, Figure S10).

### Effect of fire-suppression activity on the body temperature

The fire extinction exercise in the firehouse increased the body temperature (average increase = 1.1°C, 95% CI: 0.7, 1.4, *n* = 16, *p* < 0.001, paired t-test) immediately after the exercise. This was followed by an average decrease of 1.6°C (95% CI: -2.0, −1.1, *n* = 13, *p* < 0.001, paired t-test), compared to the temperature immediately after the exercise, measured at 60 min or more after the exercise. Following the flashover container exercise, we observed an average increase of 0.8 **°**C (95% CI: 0.6, 1.0, *n* = 8, *p* < 0.001, paired t-test) followed by an average decrease of 1.3 **°**C (95% CI: -1.8, −0.8, *n* = 7, *p* < 0.001, paired t-test), measured after 20 min and compared to the temperature immediately after the exercise. It should be noted that carryover effects cannot be ruled out as the subjects did both exercises on the same day in relatively close succession.

### Cardiovascular measurements

**Fig. 2:**
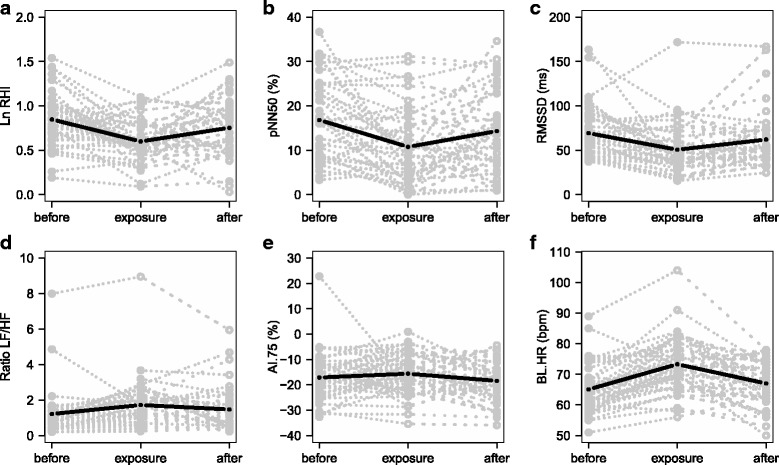
Cardiovascular endpoints in the three exposure scenarios (before and after as control measurements, and exposure measurement). Natural base log of reactive hyperemia index (one subject was eliminated due to missing data in both control measurements) (**a**), time domain heart rate variability in pNN50 (**b**) and RMSSD (**c**), frequency domain heart rate variability (**d**), augmentation index corrected for 75 bpm (**e**) and baseline heart rate (**f**). Grey symbols and dashed lines are individual results in each subject. Black line is a graphical output of the mixed effect model. pNN50, proportion of successive NN intervals differing by more than 50 milliseconds divided by total number of NN intervals; RMSSD, square root of the mean squared differences of successive NN intervals; bpm, beat per minute; ms, millisecond

Figure 2 presents the effect of exposure to firefighting on the cardiovascular endpoints. One female subject was eliminated from RHI analysis and one male subject was eliminated from HRV analysis, due to missing data for both control measurements. Exposure to firefighting was associated with decreased levels of RHI and time domain HRV. Table 2 presents the estimated changes for each of the cardiovascular measurements between different exposure scenarios showing a significant effect of exposure to firefighting as categorical variable on RHI, HRV both in time and frequency domains and in baseline heart rate. The mean baseline PAT signal amplitude was only modestly altered after the fire extinction exercise (change of −0.03%, *p* < 0.001). However adjustment for the baseline PAT signal in the statistical model did not substantially change the exposure-effect relationship of cardiovascular measurements (e.g. the percent change in RHI was decreased from −21.9% (95% CI: -32.0,-10.3) to −16.5% (95% CI: -26.1, −5.6). There was no significant difference between campaigns in the exposure-effect relationship for any of the cardiovascular measurements. There were no statistically significant relationship between LnRHI and HRV measurements and urinary 1-OHP excretion (Additional file 1: Table S6). Addition of information on self-reported allergies in the statistical model did not affect the exposure-effect relationship. Outcome average results for each exposure scenario are presented in Additional file 1: Table S5.

**Table 2:** Percent change (95% confidence interval) in outcome levels estimated by mixed effects model adjusted for sex and body mass index

Outcome	Exposure vs Before	Exposure vs After	After vs Before	Exposure vs Unexposed^a^
RHI ^b^	−21.9 (−32.0,-10.3)***	−14.3 (−25.3, −1.6)**	−8.9 (−20.7, 4.6)	−18.0 (−26.0, −9.2)***
SDNN^b^	−17.3 (−28.3, −6.4)**	−10.3 (−21.7, 1.2)	−7.9 (−18.9, 3.1)	−13.2 (−22.9, −3.6)**
pNN50^b^	−36.1 (−52.3, −19.9)***	−25.1 (−43.4, −6.8)**	−14.7 (−31.0, 1.6)	−28.6 (−45.3, −11.8)***
RMSSD^b^	−26.9 (−41.0, −12.7)***	−18.6 (−33.9, −3.4)*	−10.2 (−24.4, 4.1)	−21.5 (−33.4, −9.7)***
LF^b^	27.0 (11.3, 42.8)***	17.8 (3.7, 31.9)*	7.8 (−8.1, 23.7)	21.1 (7.9, 34.2)**
HF^b^	−15.4 (−26.2, −4.7)**	−4.4 (−16.1, 7.4)	−11.6 (−22.4, −0.7)*	−9.1 (−19.2, 1.0)
LF/HF^b^	41.4 (15.0, 67.9)**	17.1 (−3.9, 38.2)	20.7 (−6.0, 47.4)	26.4 (6.7, 46.2)**
SP	−4.4 (−8.0, −0.7)*	−0.3 (−4.2, 3.5)	−4.0 (−7.7, −0.3)*	−2.4 (−5.7, 1.0)
DP	−3.2 (−8.6, 2.3)	5.8 (−0.2, 11.8)	−8.5 (−14.0, −3.0)**	1.1 (−3.5, 5.7)
AI.75	−8.8 (−24.8, 7.3)	−15.3 (−30.2, −0.4)*	7.7 (−8.3, 23.8)	−12.2 (−26.4, 2.1)
BL.HR	12.7 (9.0, 16.3)***	9.4 (5.8, 12.9)***	3.0 (−0.7, 6.7)	11.0 (7.7, 14.3)***

*RHI* reactive hyperemia index, *SDNN* standard deviation of all NN intervals, *pNN50* proportion of successive NN intervals differing by more than 50 milliseconds divided by total number of NN intervals, *RMSSD* square root of the mean squared differences of successive NN intervals, *LF* power in low frequency range (0.04–0.15 Hz) in ms^2^, *HF* power in high frequency range (0.15–0.4 Hz) in ms^2^, *LF/HF* ratio LF(ms^2^)/HF(ms^2^), *SP* systolic blood pressure (mmHg), *DP* diastolic blood pressure (mmHg), *AI.75* augmentation index corrected for 75 bpm, *BL.HR* baseline heart rate (bpm)

Results are percent change from the mixed effect model in Fig. 1 except for RHI where percent change was obtained directly from the effect estimate due to the logarithmic transformation. The data are based on 43 individuals with measurements in both fire extinction exercise and control exposure condition (measurements of control exposure condition were missing for one subject in RHI and heart rate variability outcomes)

*,**,*** Significantly different (*p* < 0.05, *p* < 0.01 and *p* < 0.001 respectively)

^a^ Unexposed corresponds to the mean between “Before” and “After” for each subject

^b^ One subject was eliminated due to missing data in both control measurements

Table 3 presents the average effect change for each of the cardiovascular endpoints between exposure and unexposed situations for the two different types of fire: wood and wood with mattresses and electrical cords. The results show no difference between the two different types of fire for our primary outcomes, except for blood pressure, where a statistically significant difference was observed.

**Table 3:** Within-subject effect change between exposure and unexposed situations for two different types of fires: wood pallets and wood pallets with mattresses and electrical cords

Outcome	Difference^a^with pallet fuel	Difference^a^with mixed fuel	Welch t-test *p*-value
LnRHI	−0.1 ± 0.3	−0.3 ± 0.3	0.166
SDNN	−9.3 ± 24.9	−9.8 ± 22.2	0.942
pNN50	−0.05 ± 0.1	−0.04 ± 0.1	0.879
RMSSD	−14.2 ± 29.8	−14.1 ± 22.9	0.991
LF	12.8 ± 71.0	56.6 ± 74.2	0.075
HF	−18.7 ± 71.0	−15.1 ± 54.7	0.857
LF/HF	0.2 ± 0.7	0.5 ± 1.0	0.174
SP	−8.2 ± 12.4	1.6 ± 11.8	0.013
DP	−3.3 ± 7.2	3.9 ± 10.5	0.012
AI.75	2.9 ± 7.8	1.6 ± 9,1	0.614
BL.HR	6.2 ± 6.7	8.1 ± 7.8	0.400

*LnRHI* natural logarithm of the reactive hyperemia index, *SDNN* standard deviation of all NN intervals, *pNN50* proportion of successive NN intervals differing by more than 50 milliseconds divided by total number of NN intervals, *RMSSD* square root of the mean squared differences of successive NN intervals, *LF* power in low frequency range (0.04–0.15 Hz) in ms^2^, *HF* power in high frequency range (0.15–0.4 Hz) in ms^2^, *LF/HF* ratio LF(ms^2^)/HF(ms^2^), *SP* systolic blood pressure (mmHg), *DP* diastolic blood pressure (mmHg), *AI.75* augmentation index corrected for 75 bpm, *HR* baseline heart rate (bpm). Values are mean ± SD

^a^Average difference between the exposed and unexposed situations within each subject

## Discussion

The present study showed that participation in fire extinction exercise did not cause PM exposure during firefighting using the PPE with self-contained breathing apparatus, whereas PM exposure occurred when the self-contained breathing apparatus was taken off in areas considered safe. Participation in firefight training resulted in exposure to PAHs in terms of increased urinary excretion of 1-OHP, increased body temperature and with cardiovascular risk markers in terms of both decreased microvascular function and changed HRV.

In the present study, there was no association between urinary excretion of 1-OHP and cardiovascular risk markers. Urinary excretion of 1-OHP has been established as a reliable biomarker of internal dose of PAHs in populations exposed to urban air pollution [21]. Our results demonstrate that the subjects were exposed to PAHs, although we did not appoint sources of PAHs in the present study. PAH exposure occurs both by inhalation of PM and by dermal exposure to soot [22]. Our results indicate that the exposure to PAH is a weak predictor of cardiovascular risk markers as compared to other risk factors such as physical exhaustion and heat. Both of these alter blood flow. Nevertheless, it should be noted that the firefighting exercises encompassed simultaneous exposure to smoke, heat and physical activity. It is not possible to separate the effect of smoke exposure on cardiovascular endpoints from that of heat and physical activity in the present study. It is possible that the observed short-term vascular effects predominantly reflects effects related to increased blood flow in order to ameliorate peripheral built-up of waste products from the physical exercise and reduce the core body temperature related to the last of the smoke diving exercises in the 3-day course. We did not observe any difference in the microvascular function and HRV between fires with or without mattresses and electrical cords. In parallel to the biomarkers of cardiovascular risk described in the present study, PAH exposure on skin and biomarkers of inflammation and genotoxicity in blood were assessed for the 53 study subjects [23]. Firefighting did not affect blood levels of C-reactive protein, serum amyloid A, IL6 and IL8 concentrations, whereas there was increased level of oxidatively damaged DNA (i.e. formamidopyrimidine DNA glycosylase sensitive sites measured by the comet assay) in peripheral blood mononuclear cells compared to the mean of two control measurements performed 2 weeks before and 2 weeks after the fire fighting course [23]. The results suggest systemic oxidative stress, which is linked to cardiovascular disease.

The assessment of ambient air levels of PM indicated high concentrations inside and outside the firehouse. PM inside the firehouse came from the fire, whereas outdoor exposure represents dispersion of smoke from the firehouse and exhaust from a diesel-driven fire truck near the entrance of the firehouse. Other studies have demonstrated elevated levels of 1-OHP in subjects participating in firefighting exercises using diesel as fuel [24] and real-life fires [25]. Firefighting activities on wood fire have yielded rather low urinary levels of 1-OHP, whereas other types of urinary hydroxylated PAHs have been elevated post-exposure [26–28]. We did not obtain information on the total personal exposure to PM because of limited number of samplers and because we chose to assess PM exposure during firefighting while wearing PPE for more subjects instead of assessing whole day exposure for one or two subjects. During firefighting there was little PM inhalation exposure because the self-contained breathing apparatus was a highly efficient barrier toward particles. Pulmonary exposure was only observed when the subjects were not wearing the full PPE. The exposure assessment indicated substantial PM exposure in the areas considered safe.

We found a decreased microvascular function, measured by RHI, after the fire extinction exercise compared to the no-exposure scenario. A decreased microvascular function, using EndoPAT, has previously been described in exposure studies on air pollution particles in susceptible groups such as elderly [18, 29], whereas mixed results were reported for young and healthy subjects [30, 31]. Likewise, short-term controlled exposure studies on diesel exhaust have shown associations with reduced vasodilatory response [32]. However, short-term controlled exposure to high concentrations of wood smoke, i.e. several hundred micrograms per cubic meter, have demonstrated unaltered or even increased vasodilation response [12, 13]. Low level of flow-mediated vasodilation corrected for shear stress is a risk factor to cardiovascular disease in firefighters; other additive risk factors are Framingham risk score and carotid intima-media thickness [33].

Altered HRV was manifested in both time and frequency domains. The fire extinction exercise was associated with decreased time domain HRV measures (SDNN, pNN50 and RMSSD), reduced high frequency components (HF), increased low frequency components (LF) and increased LF/HF ratio. Overall, it indicates an imbalance in the autonomic activation of the heart with reduced vagal activity and increased sympathetic activity. A meta-analysis has recently shown reduced measures of HRV in humans after exposure to particulate air pollution [9], whereas a review of panel studies concluded that the studies did not convincingly show inverse associations between ambient air PM_2.5_ concentrations and HRV [34]. Two controlled studies reported no association between short-term diesel exhaust (100–300 μg/m^3^ for 2 h) and HRV [35, 36]. However, a short-term controlled exposure to wood smoke study (314 μg/m^3^ for 3 h) showed reduced HRV and increased heart rate during a 1-h post-exposure period [14]. Reduced HRV has been shown to be associated with increased risk of a first cardiovascular event in people without cardiovascular diseases [37].

Despite the demand for physical fitness, firefighters as a group seem to harbour several risk factors for cardiovascular diseases. In a recent study on young career firefighters (<45 years), increased risk of sudden cardiac death was found to be largely attributed to obesity, hypertension and smoking [38]. Therefore, to avoid effect modification due to lifestyle factors, we used young and non-smoking conscripts who were generally healthy in our study. It is generally acknowledged that exposure to air pollution has an immediate effect, e.g. precipitation of myocardial infarction, and a chronic effect related to progression of atherosclerosis. Consequences of this difference in effect are apparent in the risk estimates from short-term and long-term exposure in epidemiological studies, whereas a time-integrated exposure metric suggests a monotonic exposure-effect relationship [39]. Our study is by design revealing short-term effects on both the vasculature and myocardium. The observation suggests that a reduced microvascular vasodilation response would be associated with increased peripheral resistance and progression toward hypertension and left ventricular cardiac overload due to backward failure. Indeed, HRV is reduced in patients with hypertension [40]. Increased physical workload, heat and dehydration also can be independent risk factors for increased risk of mortality from coronary heart disease among on-duty firefighters, whereas conditional risk factors for cardiovascular disease such as obesity, dyslipidemia, hypertension and diabetes may put certain subjects in high-risk category for sudden cardiac death.

## Conclusions

In the present study, exposure of human volunteers following a 3-day firefighting training program with various types of exercises in a firehouse was associated with altered cardiovascular effects in terms of decreased microvascular function and altered HRV. The subjects were very efficiently protected against pulmonary PM exposure when using the full personal protective equipment including the self-contained breathing apparatus. Significant PM exposure was observed when the subjects took off their self-contained breathing apparatus in areas considered safe. Fire extinction exercises were associated with increased urinary 1-OHP levels indicating exposure to PAH. However, the association between urinary excretion of 1-OHP and cardiovascular effects was not statistically significant in models that included smoke exposure as categorical variable. Physical activity and heat are also conditions that occur during the fire extinction exercise, which alter blood flow. Thus, the altered cardiovascular responses after fire extinction exercises are most likely due to complex effects from PM exposure, physical exhaustion and increased core body temperature.

## Additional file

Additional file 1: Supplementary material. (DOC 4338 kb)

## Abbreviations

1-OHP: 1-hydroxypyrene
AI: Augmentation index
BL.HR: Base line heart rate
BMI: Body mass index
CI: Confidence interval
DP: Diastolic blood pressure
HF: High frequency
HPLC: High performance liquid chromatography
HRV: Heart rate variability
LF: Low frequency
PAH: Polycyclic aromatic hydrocarbons
PAT: Peripheral arterial tonometry
PM: Particulate matter
pNN50: proportion of normal-to-normal intervals differing by more than 50 miliseconds
PPE: Personal protective equipment
RHI: Reactive hyperemia index
RMSSD: Root mean square of the successive differences
SDNN: Standard deviation of normal-to-normal intervals
SP: Systolic blood pressure
