# Supplementary material for: Cardiovascular health effects following exposure of human volunteers during fire extinction exercises
Source: Environ Health. 2017 Sep 6;16:96. doi: 10.1186/s12940-017-0303-8 (PMC5588677; doi:10.1186/s12940-017-0303-8)
Supplement: Supplementary file 1 — Supplementary material. (DOC 4338 kb) [file 12940_2017_303_MOESM1_ESM.doc]

**ADDITIONAL FILE 1**

**Supplementary material to the article:**

**Cardiovascular health effects following exposure of human volunteers during fire extinction exercises**

Maria Helena Guerra Andersen^1,2^, Anne Thoustrup Saber^2^, Peter Bøgh Pedersen^3^, Steffen Loft^1^, Åse Marie Hansen^2,4^, Ismo Kalevi Koponen^2^, Julie Elbæk Pedersen^5^, Niels Ebbehøj^5^, Eva-Carina Nørskov^3^, Per Axel Clausen^2^, Anne Helene Garde^2,4^, Ulla Vogel^2,6^, Peter Møller^1^

^1^Department of Public Health, Section of Environmental Health, University of Copenhagen, Øster Farimagsgade 5A, DK-1014 Copenhagen K, Denmark; ^2^The National Research Centre for the Working Environment, Lersø Parkalle 105, DK-2100 Copenhagen Ø, Denmark; ^3^Danish Technological Institute, Teknologiparken, Kongsvang Allé 29, DK-8000 Aarhus C, Denmark; ^4^Department of Public Health, Section of Social Medicine, University of Copenhagen, Øster Farimagsgade 5A, DK-1014 Copenhagen K, Denmark; ^5^Department of Occupational and Environmental Medicine, Bispebjerg Hospital, Bispebjerg Bakke 23, DK-2400 Copenhagen NV, Denmark; ^6^Department of Micro- and Nanotechnology, Technical University of Denmark, DK-2800 Kgs. Lyngby, Denmark

Contents

[**I.** **DESCRIPTION OF THE EXPOSURE SETTING** 2](#__RefHeading___Toc477252692)

[**II.** **URINARY 1-HYDROXYPYRENE PER CAMPAIGN** 14](#__RefHeading___Toc477252693)

[**III.** **AVERAGE VALUES FROM THE THREE EXPOSURE SCENARIOS** 15](#__RefHeading___Toc477252694)

[**IV.** **ESTIMATE LEVELS USING 1-OHP AS CONTINUOS EXPOSURE VARIABLE** 16](#__RefHeading___Toc477252695)

# **DESCRIPTION OF THE EXPOSURE SETTING**

The exposure setting is depicted in supplementary figure S1. The drawing does not depict the true scale of the building. We measured particle concentrations at different locations and with different equipment. The measurements were designed to document exposure at different hotspots, whereas it was not possible to obtain a detailed exposure of all individuals. As different particle measurement equipment was used in different locations, it is generally not possible to compare measurements from different locations unless the same equipment has been used in time-series measurements. Supplementary table S1 summarises the particle measurement equipment, locations and certain specifications of the measurements.

**Measurement of air concentration of particulate matter (PM) in the firehouse***.* Supplementary figure S2 shows a time-series measurement of the mass concentration of particles in a room adjacent to the room with the fire in the firehouse (wood fire, campaign 1). It was not possible to measure the PM mass concentration in the room with fire in that specific exercise. The PM mass concentrations in the adjacent room were measured with a DustTrack. The PM mass concentrations in the adjacent room were characterized by high peaks, which coincided with ignition and fire. There were lower concentrations when the fires were extinguished, before new teams entered the firehouse (new ignitions approximately at 8h45, 9h15 and 9h30). In the adjacent room, the average total PM mass concentration over the exposure period was 550 µg/m^3^. Measurements of the mass concentration on the 1^st^ floor landing (the floor above the fire extinction exercise area) was constantly high (average total PM mass concentration = 32 mg/m^3^).

**Measurement of personal exposure.** Supplementary figure S3 depicts an example of a time-series measurement of the particles for one person before, during and after a fire suppression exercise in the firehouse (miniature diffusion size classifier DISCmini). As can be seen, the person is exposed to high PM number concentration before and after the smoke diving exercise, whereas there is no exposure during the exercise when the person was wearing the personal protective equipment (PPE) including the self-contained breathing apparatus (mask). Supplementary figure S8 shows the PPE including the self-contained breathing apparatus. Supplementary table S2 shows values from three different smoke-diving exercises that particular day. The exposure without the mask (i.e. exposure during briefing and debriefing periods) varied substantially between individuals, whereas the mask effectively prevented inhalation of particles. The particle size inside the mask indicates that the particles were very small, but the low particle number concentration makes the calculation of the particle size uncertain. Figure S9 shows the inlet position of the tube to the DISCmini inside the self-contained breathing apparatus.

**Measurement of particle concentrations at different locations**. Supplementary table S4 shows the measurements with a particle monitor in the classroom or room of biological measurements. Supplementary figure S4 shows a time-series measurement of the particle number concentration immediately outside the firehouse, corresponding to location 1 on supplementary figure S1. The mean particle number over the measurement period was 48 297 particles/cm^3^. In comparison, the mean particle number concentration in busy streets of Copenhagen, Denmark is approximately 30 000 particles/cm^3^.

Supplementary figure S5 shows a time-series of the particle number concentration inside the firehouse (corresponding to location 1 on supplementary figure S1), using a Nanoscan scanning mobility particle sizer (Nanoscan SMPS). The same instrument was also used for measurements on the two preceding days of the 3-day smoke-diving exercise. Supplementary table S3 shows the particle number concentration and average particle size. There is substantial inter-day variation in the exposure. It is not possible to appoint exposures to specific exercises or persons and it should be noted that the tasks became increasingly more demanding during the 3-day course as the skills of the participants improved.

**Table S1.** Equipment used for particle measurement inside the firehouse (1), information zone (2), outside the building (4), flashover container (5) and building used for blood sampling and other biological measurements (6).

| **Equipment** | **Location** | **Particle size** | **Notes** |
| --- | --- | --- | --- |
| **Nanoscan SMPS model 3910 (TSI, USA)** | Stationary (location 1) | 10-350 nm | Measures number concentration of ultrafine particles (UFP) in 13 size bins, giving rise to a particle size distribution (PSD) |
| **P-Trak, Ultrafine Particle Counter 8525 (TSI, USA)** | Stationary (location 1) | 20-1 000 nm | Measures number concentration of ultrafine particles (UFP) |
| **DustTrak DRX, model 8533 (TSI, USA)** | Stationary (location 1) | ~0.1-15 µm | Measures particulate matter (PM) in size fractions PM_1_, PM_2.5_, PM_4_, PM_10_, PM_total_. We have used the total PM concentration in the study. |
| **DiSCmini (Testo, Titisee-Neustadt, Germany)** | Portable  (location 3, 4, 5)  Stationary (location 2) | 10-700 nm | Measures number concentration and size of ultrafine particles (UFP) and calculates lung deposited surface area (LDSA) of particles. The equipment was placed on a person in a bag underneath the PPE. A Tygon tube connected the instrument to the inside of the self-contained breathing apparatus (mask), making it possible to measure the particle exposure as close as possible to the breathing zone. The measurements consists of time-series with periods of measurements inside the mask and periods when the conscript took off the mask before/after the exercise. |
| **Aerasense NanoTracer (Oxility, Eindhoven, the Netherlands)** | Stationary (location 6) and stationary at control measurements | 10-300 nm | Portable device designed for continuous measurements of ultrafine particles (UFP) |

**Table S2.** Mean particle number concentration and particle size in personal monitor (DiSCmini) during smoke-diving (with mask^a^) and before/after (without mask^b^) (n=3)

| **Measurement and time period** | **With mask** | | **Without mask** | |
| --- | --- | --- | --- | --- |
|  | Mean (max) [#/cm³] | Particle diameter [nm] | Mean (max) [#/cm³] | Particle diameter [nm] |
| **1 (9:00-9:45)** | 876 (3 309) | <10^c^ | 54 799 (282 193) | 98 |
| **2 (9:45-10:20)** | 799 (3 111) | <10^c^ | 17 640 (136 543) | 41 |
| **3 (12:15-13:10)** | 866 (27 778)^d^ | <10^c^ | 244 387 (2 318 282) | 51 |

^a^The subjects were wearing personal protection equipment, including clothing, gloves, helmet and self-contained breathing apparatus. The DiSCmini measured particles inside the self-contained breathing apparatus (mask), close to the breathing zone of the subjects (figure S9).

^b^ The subjects removed the mask at “safe” locations where the smoke level was considered low (i.e. “without mask”).

^c^The particle size inside the PPE indicates that the particles were very small, but the low particle number concentration makes the calculation of the particle size uncertain.

^d^ It was found, that the mask was not placed and adjusted correctly during firefighting, giving rise to the high value of maximum particle number concentration.

**Table S3.** Particle number concentration and particle size in stationary samples (Nanoscan SMPS). Number concentration dN/dlogDp, 13 size bins. Mean diameter given by arithmetic mean of number weighted concentration per size channel. The measurement was carried out at location 1 in supplementary figure S1

| **Day of exposure** | **Mean particle number (#/cm^3^)** | **Mean Diameter (nm)** |
| --- | --- | --- |
| **1** | 634 853 | 78 |
| **2** | 378 671 | 111 |
| **3** (day of sample collection ) | 102 173 | 97 |

**Table S4.** Particle number concentration and particle size measured in the classroom where the conscripts were waiting or in the room of biological measurements with a Nanotracer device. The measurements were carried out at a room in the residential place where the control visits (before and after) were done and at location 6 in supplementary figure S1 (exposure day)

| **Campaign** | **Exposure scenario** | **Location** | **Mean (max) [#/cm³]** | **Mean particle diameter [nm]** |
| --- | --- | --- | --- | --- |
| **1** | Control before | Residential place | 42 924 (1 197 350)^a^ | 37 |
| **1** | Control after | Residential place | 13 371 (536 927) | 54 |
| **2** | Control before | Residential place | 26 226 (102 021) | 35 |
| **2** | Exposure day | Location 6 (figure S1) | 7 290 (10 729) | 47 |
| **2** | Control after | Residential place | 2 079 (3 160) | 90 |
| **3** | Control before | Residential place | 7 010 (11 100) | 52 |
| **3** | Control after | Residential place | 3 048 (4 328) | 79 |
| **4** | Control before | Residential place | 9 374 (11 414) | 65 |
| **4** | Exposure day | Location 6 (figure S1) | 4 864 (7 108) | 68 |
| **4** | Control after | Residential place | 5 550 (7 826) | 85 |

^a^ The pick corresponds to a brief event where the instructor burned something in the classroom

**Figure S1.** Drawing of the building where the fire extinction exercises took place. Pictures of the firehouse and entrance area are shown in figure S6 and S7.


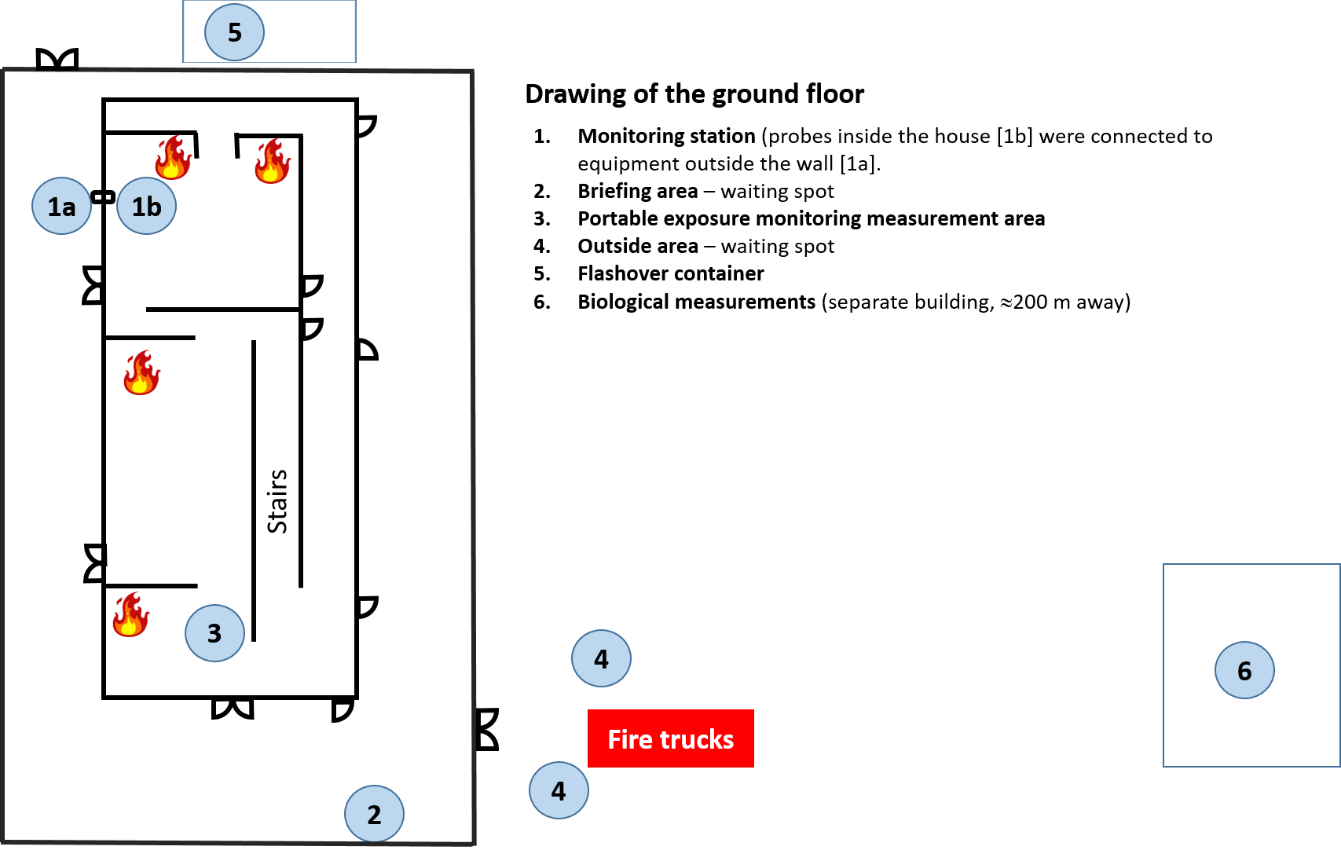


**Figure S2**. Measurement of aerosol particle mass concentration in an adjacent room to the fire in the firehouse (campaign 1, wood fire). Measured with DustTrak.


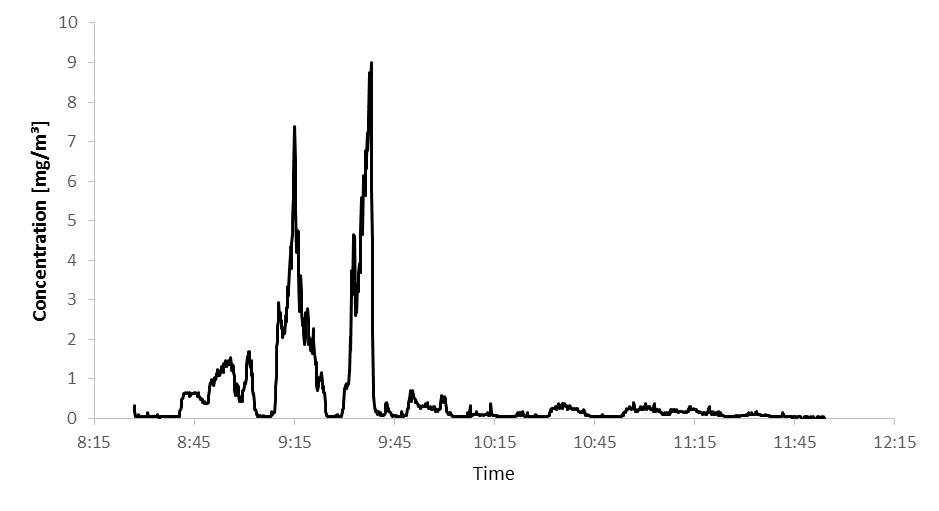


**Figure S3**. Time-series for one person measured in the breathing zone with and without the personal protective equipment, including self-contained breathing apparatus (mask) during a day where electrical cables and mattresses were burnt in the house (campaign 3). Lines represent the particle number concentration (black) and the mean particle diameter (red). Measured with DISCmini.


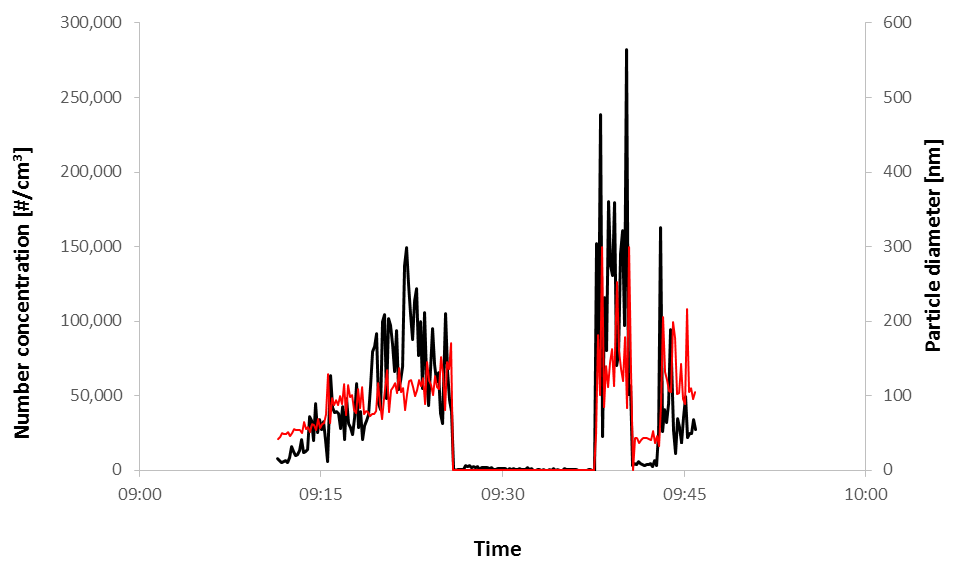


*Instruction (location 2)*

*Without mask*

*Smoke diving*

*Exercise (location 3) Wearing mask*

*Instruction (location 2 and 4)*

*Without mask*

**Figure S4**. Particle number concentrations immediately outside the firehouse on a day with combustion of wood. The particle number concentration is a stationary measurement with P-Track (campaign 1, wood fire).


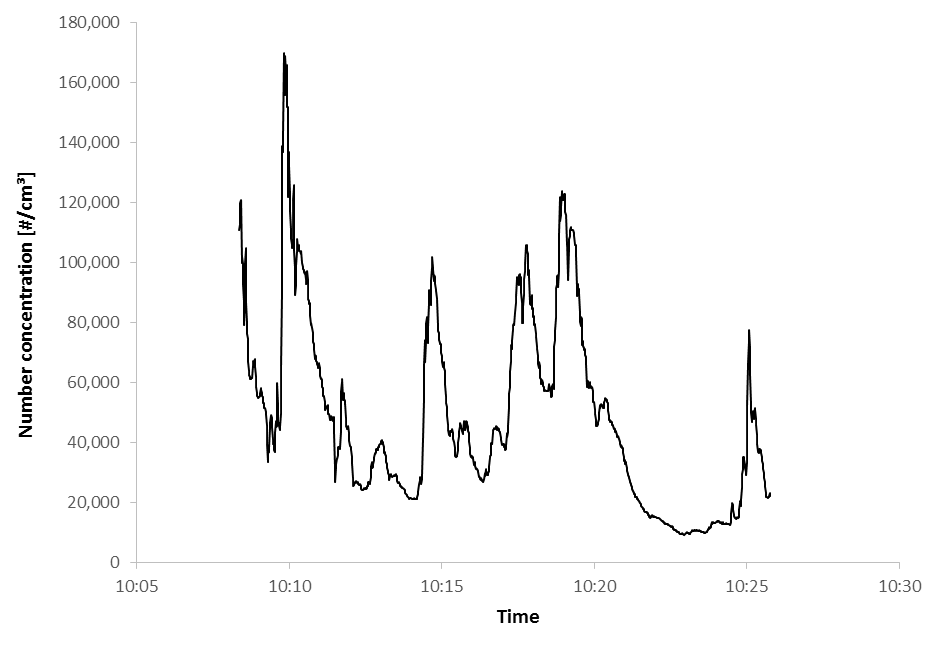


**Figure S5**. Time-series of the particle number concentration inside the firehouse (corresponding to location 1 on supplementary figure S1), using a scanning mobility particle sizer (Nanoscan SMPS)


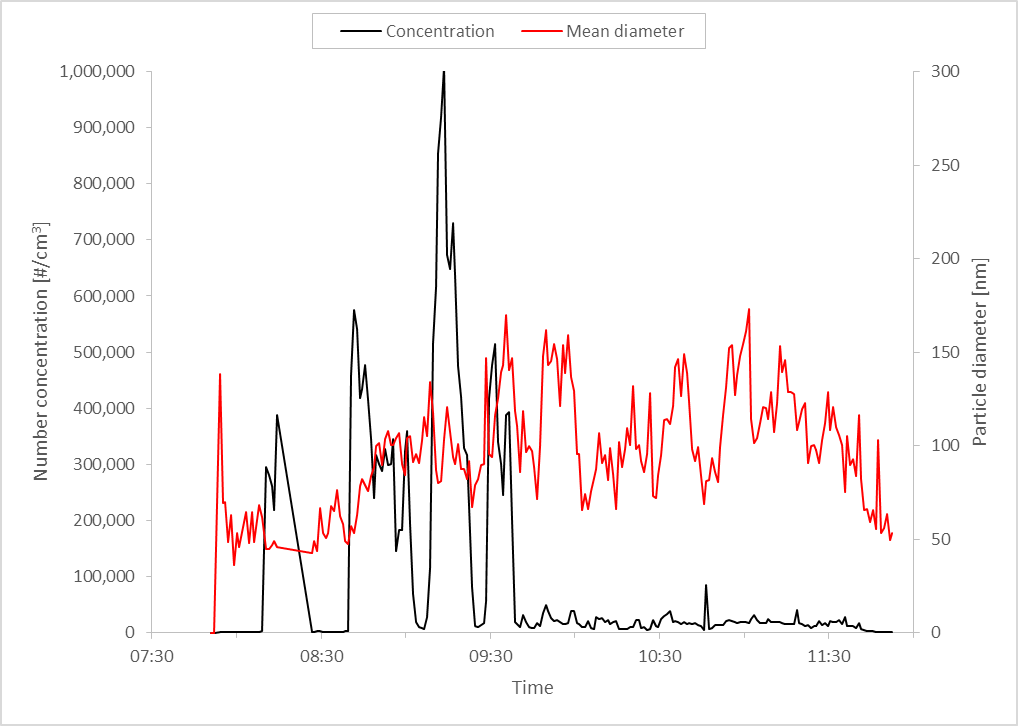


**Figure S6**. Two-floor building where the fire extinction exercises took place.

**
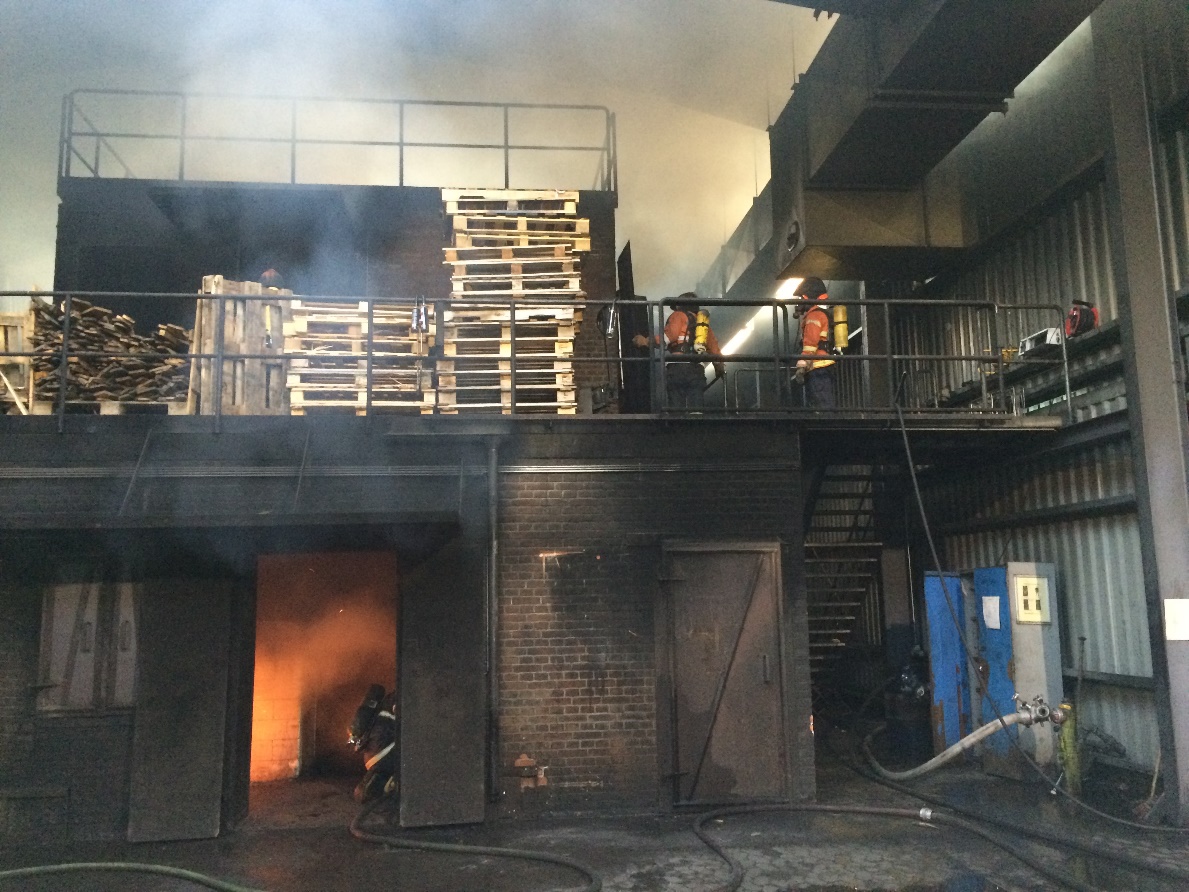
**

**Figure S7**. Outside area marked as number 4 in figure S1

**
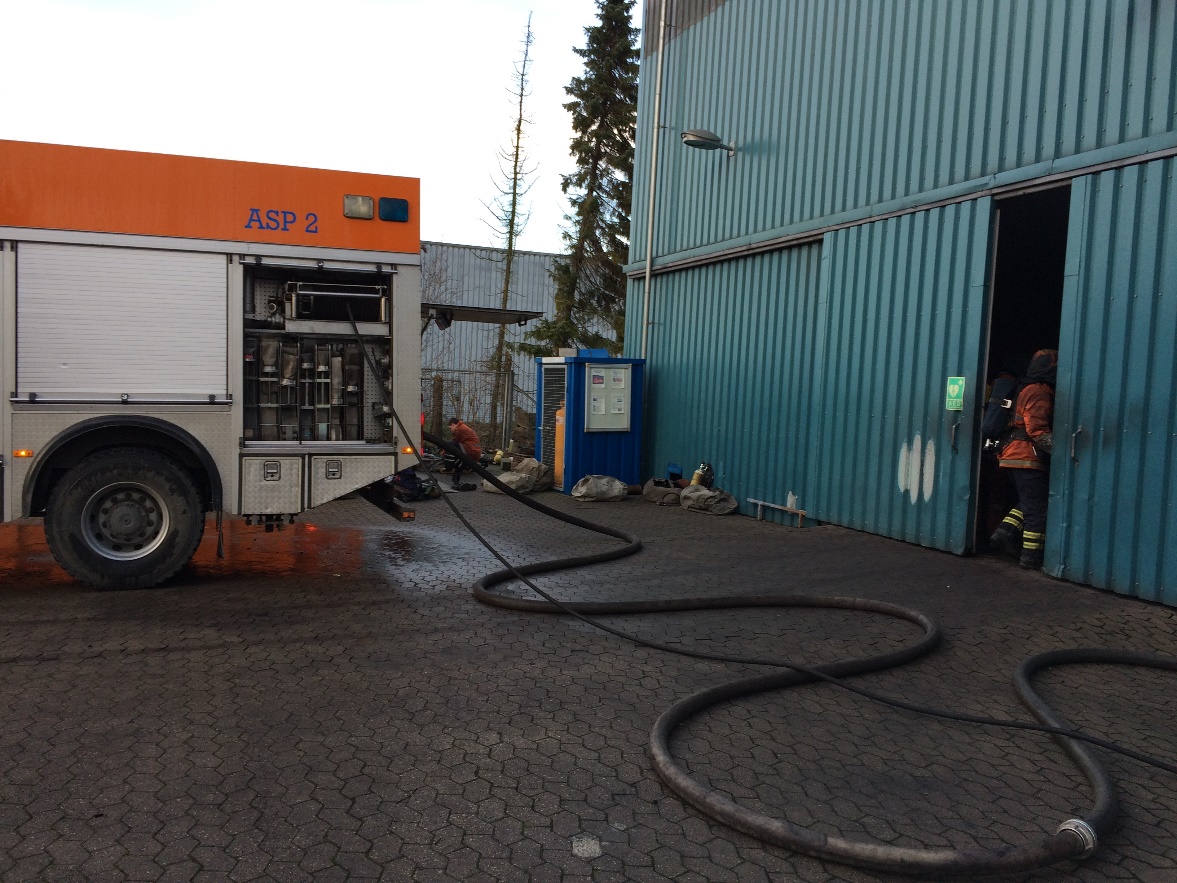
**

**Figure S8**. Team of two conscripts and one instructor starting the fire extinction exercise

**
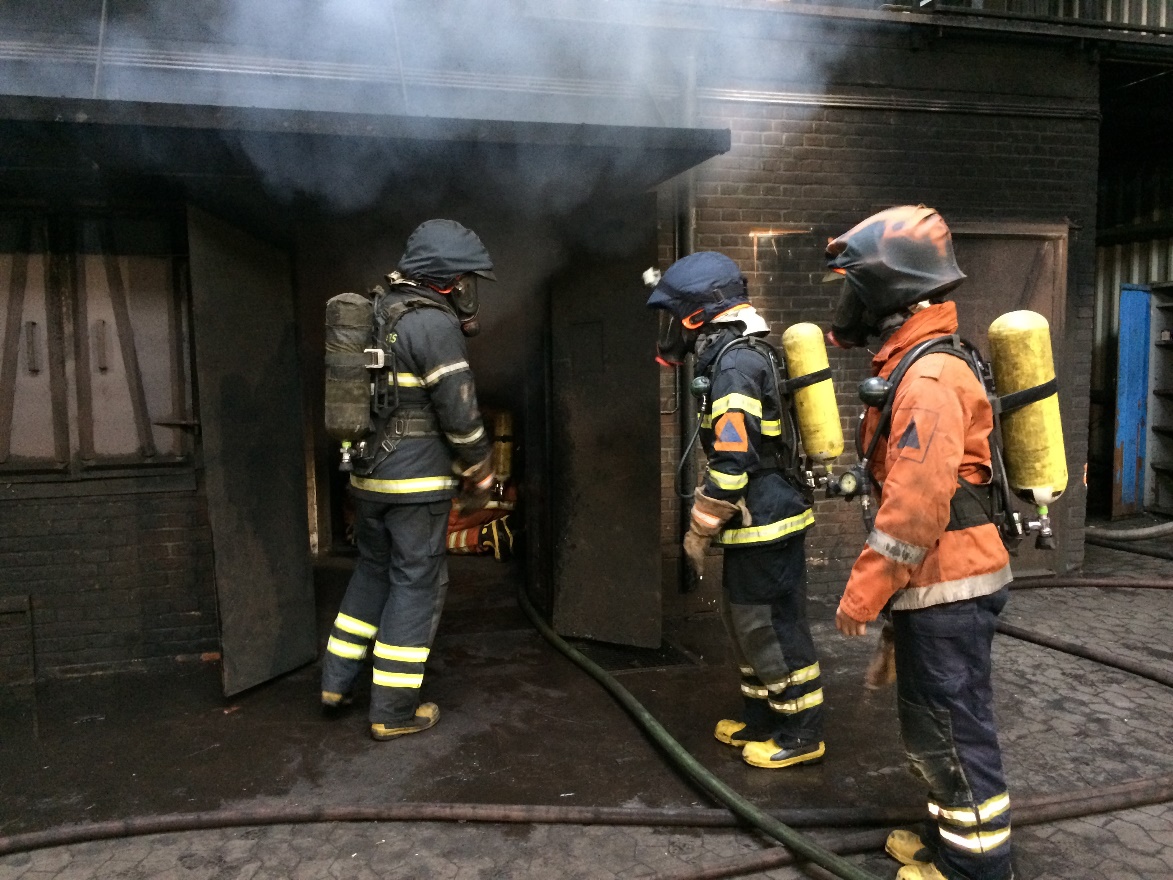
**

**Figure S9**. DISCmini inlet position inside the self-contained breathing apparatus

**
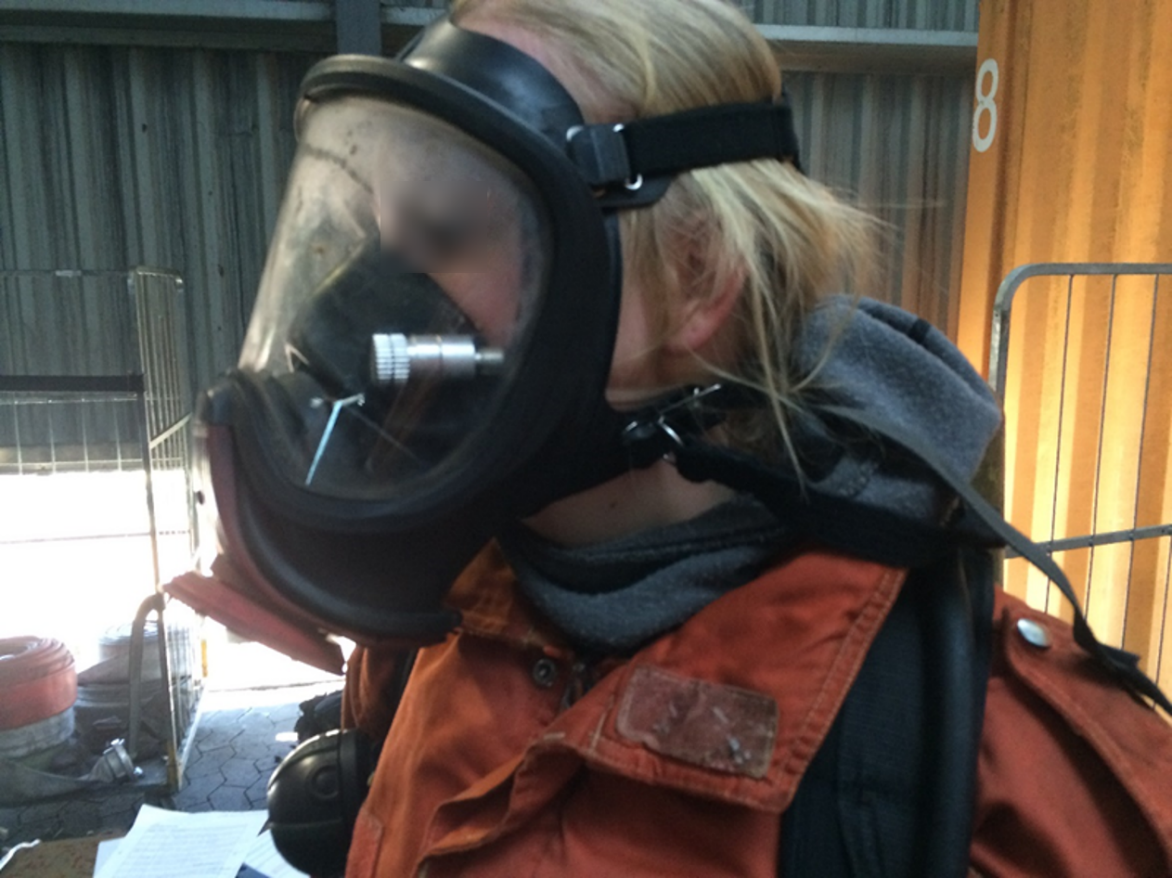
**

# **URINARY 1-HYDROXYPYRENE PER CAMPAIGN**

**Figure S10.** Creatinine-adjusted urinary concentration of 1-hydroxypyrene in the three exposure scenarios per campaign (four campaigns). Campaign 1 and Campaign 2 (wood), Campaign 3 and Campaign 4 (wood, mattresses and electrical cords). “Before” and “after” are periods without exposure, and “exposure” is after the fire extinction exercise.


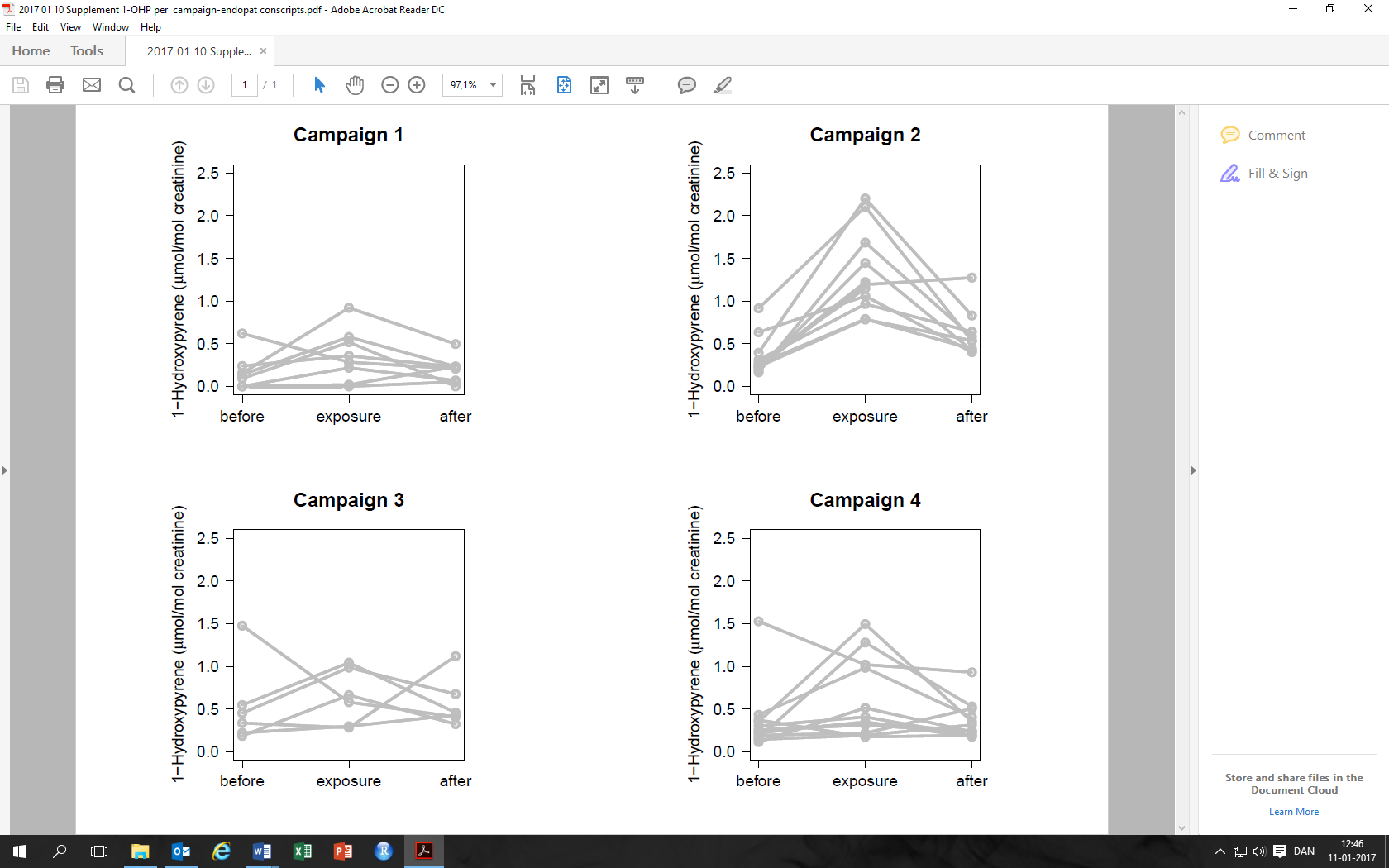


# **AVERAGE VALUES FROM THE THREE EXPOSURE SCENARIOS**

**Table S5.** Average (±SD) of the outcome levels from three exposure scenarios (Before, Exposure and After)

| **Outcome** | **Before** | **Exposure** | **After** |
| --- | --- | --- | --- |
| **Ln.RHI** | 0.8 ± 0.3 (n=41) | 0.6 ± 0.2 (n=42) | 0.8 ± 0.3 (n=42) |
| **SDNN** | 76.7 ± 24.3 (n=37) | 62.2 ± 28.7 (n=42) | 69.2 ± 28.7 (n=41) |
| **pNN50** | 17.4 ± 10 (n=37) | 10.8 ± 10 (n=42) | 14.3 ± 10 (n=41) |
| **RMSSD** | 70.9 ± 30.7 (n=37) | 50.6 ± 28.3 (n=42) | 61.9 ± 32.6 (n=41) |
| **LF** | 169.0 ± 97.5 (n=37) | 219.3 ± 92.3 (n=42) | 186.3 ± 91.2 (n=41) |
| **HF** | 197.2 ± 73.5 (n=37) | 164.5 ± 72.2 (n=42) | 171.0 ± 72.4 (n=41) |
| **LF/HF** | 1.15 ± 1.4 (n=37) | 1.72 ± 1.4 (n=42) | 1.48 ± 1.3 (n=41) |
| **SP** | 116.5 ± 11.4 (n=43) | 111.4 ± 12.1 (n=43) | 111.8 ± 17.2 (n=43) |
| **DP** | 66.4 ± 9.5 (n=43) | 64.3 ± 8.0 (n=43) | 60.7 ± 11.1 (n=43) |
| **AI.75** | -17.1 ± 9.6 (n=43) | -15.6 ± 8.4 (n=43) | -18.4 ± 7.6 (n=43) |
| **BL.HR** | 65.1 ± 8.0 (n=43) | 73.3 ± 9.2 (n=43) | 67.0 ± 7.4 (n=43) |
| **1-OHP** | 0.35 ± 0.3 (n=36) | 0.79 ± 0.5 (n=36) | 0.44 ± 0.3 (n=34) |

Ln.RHI, natural logarithmic of reactive hyperemia index (RHI); SDNN, standard deviation of all NN intervals; pNN50, proportion of successive NN intervals differing by more than 50 milliseconds divided by total number of NN intervals; RMSSD, square root of the mean squared differences of successive NN intervals (ms); LF, power in low frequency range (0.04-0.15 Hz) in ms^2^; HF, power in high frequency range (0.15-0.4 Hz) in ms^2^; LF/HF, ratio LF(ms^2^)/HF(ms^2^); SP, systolic blood pressure; DP, diastolic blood pressure; AI.75, augmentation index corrected for 75 bpm; HR, base line heart rate (bpm); 1-OHP, urinary excretion of 1-hydroxypyrene adjusted for excreted creatinine concentration (µmol/mol creatinine).

# **ESTIMATE LEVELS USING 1-OHP AS CONTINUOS EXPOSURE VARIABLE**

**Table S6.** Estimate (±SE) in outcome levels by mixed effects model with creatinine adjusted urinary excretion of 1-hydroxypyrene, sex and body mass index as fixed effects and by subject intercepts as random effect.

| **Outcome** | **Estimate (±SE)** | **p-value** |
| --- | --- | --- |
| **Ln.RHI** | -0.143691 (± 0.069) | 0.111 |
| **SDNN** | -4.932 (± 5.499) | 0.761 |
| **pNN50** | -0.022056 (± 0.019) | 0.592 |
| **RMSSD** | -8.9024 (± 6.560) | 0.452 |
| **LF** | 26.841 (± 17.186) | 0.327 |
| **HF** | -16.893 (± 13.822) | 0.543 |
| **LF/HF** | 0.25763 (± 0.209) | 0.536 |
| **SP** | -1.8517 (± 2.536) | 0.856 |
| **DP** | 0.2677 (± 2.131) | 0.999 |
| **AI.75** | -0.6736 (± 1.798) | 0.978 |
| **BL.HR** | 4.3142 (± 1.738) | 0.042 |

Ln.RHI, natural logarithmic of reactive hyperemia index (RHI); SDNN, standard deviation of all NN intervals; pNN50, proportion of successive NN intervals differing by more than 50 milliseconds divided by total number of NN intervals; RMSSD, square root of the mean squared differences of successive NN intervals (ms); LF, power in low frequency range (0.04-0.15 Hz) in ms2; HF, power in high frequency range (0.15-0.4 Hz) in ms2; LF/HF, ratio LF(ms2)/HF(ms2); SP, systolic blood pressure; DP, diastolic blood pressure; AI.75, augmentation index corrected for 75 bpm; BL.HR, base line heart rate (bpm).

The data are based on 36 individuals with creatinine-adjusted urinary 1-hydroxypyrene measurements in both fire extinction exercise and control exposure condition and measurements of control exposure in RHI.
